# 2721. Respiratory Virus Infections Following CAR T-cell Therapy: Risk Factors and Outcomes

**DOI:** 10.1093/ofid/ofad500.2332

**Published:** 2023-11-27

**Authors:** Natasha Powell, Jennifer Pisano, Michael T Czapka

**Affiliations:** University of Chicago, Chicago, Illinois; University of Chicago Hospital, Chicago, Illinois; University of Chicago Hospital, Chicago, Illinois

## Abstract

**Background:**

Chimeric antigen receptor (CAR) T-cell therapy is a novel cancer therapy which functions by engineering patients’ own cells to target a malignancy. The preparative lymphodepleting chemotherapy and cell versus tumor effect create a novel, dynamic state of immunocompromise. Respiratory viruses have been found to be the predominant late infectious complication, but little is known about specific risk factors and outcomes of these infections.

**Methods:**

We retrospectively identified adult patients who received CD19-directed CAR T-cell therapy at our center and followed them up to two years post-infusion (or until disease progression, death, or February 2022 — whichever came first). We undertook structured chart review using REDCap. Descriptive statistics were used where indicated. An adapted ordinal scale was used to assess the severity of respiratory virus infections (RVIs) (PMCID: PMC9278199).

**Results:**

We found 73 patients who met search criteria. Of those, 23 patients (32%) developed an RVI, and their baseline characteristics are listed in **Table 1**. There were 38 episodes of RVI with an average of 1.65 episodes per affected patient. The median time from infusion to RVI was 180 days. The etiologic agent for the majority of RVIs was either unknown or rhino/enterovirus (**Figure 1**). The median ordinal score for RVI severity was 1, corresponding to ambulatory management for most (**Figure 2**). However, 24% of patients required a higher level of care. In patients where serial NAATs were performed, the duration of viral shedding was a median of 33 days (IQR 32 – 39). Immediately prior to infection the median IgG was 370 mg/dl (IQR 292 – 566), ANC was 1690 cells/μL (IQR 873 – 3165), and ALC was 480 cells/μL (IQR 285 – 703). 10 (43%) patients were treated with IVIG and 4 (17%) patients received antivirals.

**Figure:**
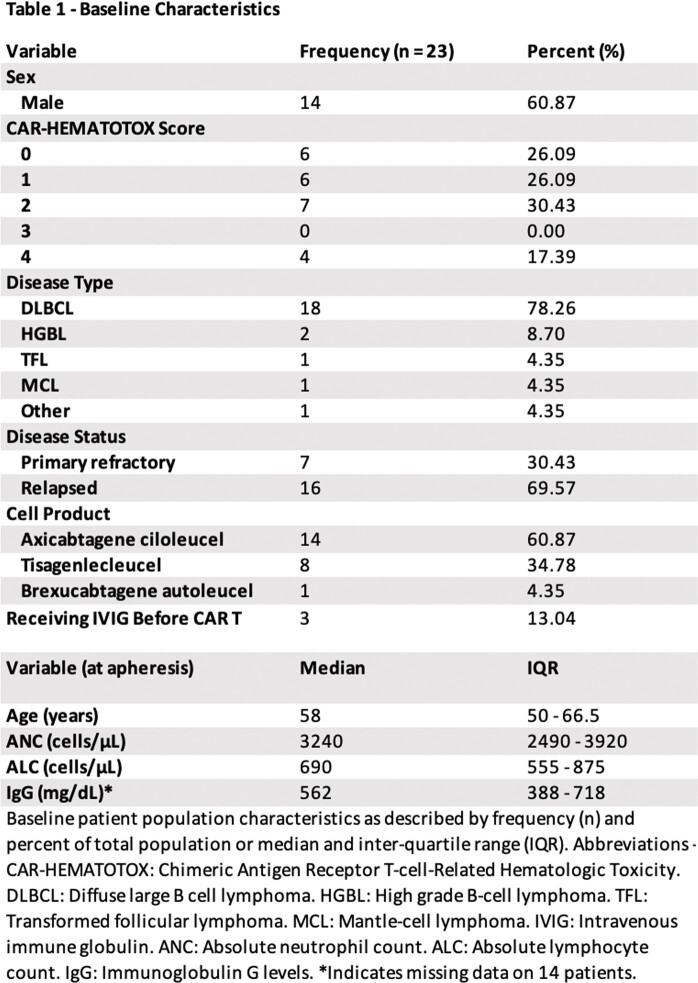

**Figure:**
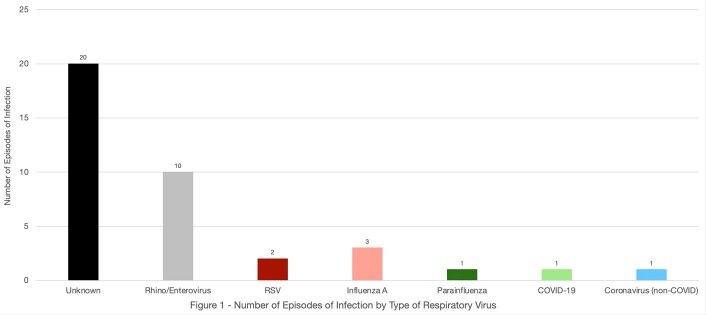

Many patients did not seek care for their RVIs or sought care outside of the Hematology/Oncology clinic. The RVI diagnosis relied on the impression of the treating physician at the time the patient presented with symptoms, or when the patient described past symptoms for which they did not present to the Hematology/Oncology clinic for care. Additionally, sometimes a full panel of respiratory viral nucleic acid amplification testing was done and negative (possibly false negative due to a collection issue or non-tested virus). Other times only COVID/FLU/RSV testing was done. Other times no respiratory virus panel was done. “Unknown” is a clinical syndrome of respiratory viral infection encompassing patients either without nucleic acid amplification testing being performed or with negative testing.

**Figure:**
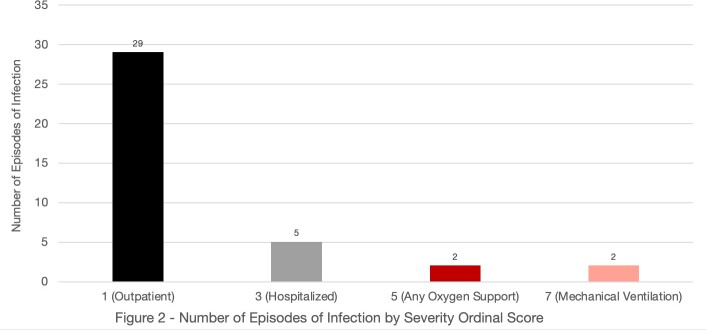

The Ordinal Scale for EHR Use* was used to rank the severity of each instance of RVI. Possible rankings included 1, 3, 5, 7, 9, or 11. None of the RVIs met the criteria for 9 (organ support) or 11 (death). *Khodaverdi M, Price BS, Porterfield JZ, Bunnell HT, Vest MT, Anzalone AJ, Harper J, Kimble WD, Moradi H, Hendricks B, Santangelo SL, Hodder SL; N3C Consortium Collaborators. An ordinal severity scale for COVID-19 retrospective studies using Electronic Health Record data. JAMIA Open. 2022 Jul 9;5(3):ooac066. doi: 10.1093/jamiaopen/ooac066. PMID: 35911666; PMCID: PMC9278199.

**Conclusion:**

There is a high burden of delayed RVI in patients who receive CAR T-cell therapy, and these infections can lead to severe illness requiring hospitalization. Clearance of virus was delayed, with duration of shedding lasting at least one month for most. Many CAR T-cell therapy patients had lower immunoglobulin G and lymphocyte counts prior to RVI. Further research is needed to understand the best treatment and prevention strategies for RVIs in this population.

**Disclosures:**

**All Authors**: No reported disclosures

